# Validity, Reliability and Interpretability of an IMU-Based System to Measure 3D Lower Limb Kinematics of Patients with Heterogeneous Gait Disorders

**DOI:** 10.3390/s26061746

**Published:** 2026-03-10

**Authors:** Lena Carcreff, Gabriel Payen, Gautier Grouvel, Mickael Cardoso-Fonseca, Fabien Massé, Stéphane Armand

**Affiliations:** 1 Kinesiology Laboratory, Geneva University Hospitals and Geneva University, 1205 Geneva, Switzerland; gautier.grouvel@unige.ch (G.G.); mickael.cfonseca@gmail.com (M.C.-F.); stephane.armand@unige.ch (S.A.); 2 Department of Physical Medicine and Rehabilitation, University Hospital of Angers, 49100 Angers, France; 3 MindMaze, 1006 Lausanne, Switzerland; gabriel.payen.gp@gmail.com (G.P.); masse.fabien@gmail.com (F.M.); 4 Center of Research on Skeletal Muscle and Movement, Geneva University Hospitals and Geneva University, 1205 Geneva, Switzerland

**Keywords:** inertial measurement units, clinical gait analysis, lower limb kinematics, concurrent validity, reliability, interpretability

## Abstract

Inertial measurement units (IMUs) represent a promising alternative to optoelectronic systems for estimating gait kinematics in less resource-intensive laboratories. However, evidence regarding the clinical interpretability of IMU-based gait analysis in pathological populations remains limited. This study aimed to evaluate the concurrent validity, reliability, and interpretability of an IMU-based method for computing 3D lower limb kinematics in asymptomatic and pathological populations. Fifty-five participants, including asymptomatic individuals (AS, n = 15), patients with cerebral palsy (CP, n = 15), and individuals with various motor disorders (OMD, n = 25), were assessed using a 7-IMU system and an optoelectronic system. Validity was quantified using root mean square error (RMSE), centered RMSE, and Pearson correlation coefficients (CCs) across 11 commonly reported lower limb kinematic outcomes. Reliability was assessed using intraclass correlation coefficients (ICCs), and interpretability was examined by comparing Gait Profile Scores (GPS) derived from both systems. Mean RMSE values were 7.1° (AS), 9.8° (CP), and 9.3° (OMD), with centered RMSE values below 3.2°. The correlation between IMU- and optoelectronic-based kinematics was good to excellent (mean CC = 0.76). Reliability ranged from moderate to excellent, and GPS showed moderate agreement between systems (CC = 0.42). These findings support the clinical validity, reliability, and interpretability of IMU-based gait kinematics across heterogeneous gait disorders.

## 1. Introduction

Tridimensional gait kinematics evaluation is crucial in some clinical situations and therapeutic decision processes. Gait kinematics is indeed an indicator of motor function and is highly valuable to assess the progression after treatment or rehabilitation over time. These quantified gait deviations are commonly assessed during a standardized examination called clinical gait analysis (CGA) [1,2] using an optoelectronic system, seen as the clinical reference and silver standard for kinematics computation. However, CGA requires expensive equipment, highly qualified personnel, and is time consuming which makes it inaccessible for some patients.

The widespread availability of inertial measurement units (IMUs) has permitted the emergence of methods to cheaply and simply estimate gait kinematics. IMU-based gait kinematics computation could indeed be possible outside of cutting edge laboratories and be performed by the clinicians themselves as the devices are simple to use and not restricted to a laboratory setting [3]. Even if the large majority of methods have been developed for asymptomatic populations, a growing interest in inertial sensors in clinical practice is observed [4,5]. Existing research has primarily focused on methodological aspects and the overall validity of the IMU-based kinematics computation [6]. Heterogeneous results were found among the studies given the diversity of methods for sensor configuration [7], sensor orientation estimation (sensor fusion process) [8] and sensor-to-segment alignment (pose estimation) [9,10]. In addition, various results were found according to the joint angle evaluated. According to Poitras et al. [11] in their review of 42 studies (including asymptomatic populations only), the root mean square errors (RMSEs) of IMUs against traditional motion capture ranged between 0.4° and 18.8° among the 12 commonly reported joint kinematic variables: pelvis, hip, knee, and ankle flexion/extension, abduction/adduction and internal/external rotation. Similarly, correlation coefficients between IMU-based and optoelectronic system-based kinematics ranged between 0.33 and 1.00 [11]. The reliability was found fair to excellent across all variables [11]. Overall, joint kinematics generally prove good to excellent validity and reliability in the sagittal and frontal planes, but this must be formally demonstrated for every new method proposed as no consensus exists on each methodological step.

Just like any other health related-patient reported outcomes (HR-PROs), biomechanical outcomes resulting from gait analysis must be reliable (free from measurement error) and valid (measures the construct(s) it purports to measure) [12]. As previously mentioned, reliability and validity aspects are commonly reported in studies aiming at proposing a new assessment tool. Notably, concurrent validity (extent of the agreement between two measures taken at the same time) is always reported. However, to be incorporated into clinical practice, gait analysis outcomes need to guarantee a high level of interpretability (degree to which one can assign qualitative meaning [12]). To the best of our knowledge, no study has assessed the interpretability of IMU-based kinematic outputs.

Therefore, the objectives of this study were to assess the concurrent validity (A), reliability (B) and interpretability (C), according to COSMIN recommendations [12], of an IMU-based method for computing 3D lower limb kinematics in asymptomatic and pathological populations with heterogeneous gait disorders. We hypothesized that the proposed method would demonstrate acceptable agreement with optoelectronic reference measures, moderate-to-excellent reliability, and clinically meaningful interpretability across populations.

The remainder of this manuscript is organized as follows: Section 2 describes participant characteristics, instrumentation, data collection, data processing, outcome selection and statistical analyses; Section 3 reports the validity, reliability, and interpretability outcomes; and Section 4 interprets findings, addresses limitations, and outlines implications for clinical practice.

## 2. Materials and Methods

### 2.1. Design of Experiment

The present study is a prospective study of concurrent validity, reliability, and interpretability The participants were all assessed once for validity and interpretability purposes. The participants who agreed to come back for a second measurement session one week later were included in the reliability part of the study. The protocol was approved by and carried out in accordance with the hospital’s institutional ethical committee (Cantonal Commission for Research Ethics of Geneva—CCER-2020-00358).

### 2.2. Participants

Three groups of participants were included in this study (from the ‘Kinemagics’ project): an asymptomatic group (AS), a cerebral palsy (CP) group, and a group of patients with various other motor disorders (OMD) (i.e., stroke survivors, patients with myopathy, with idiopathic toe walking, club foot, etc.). In each group, three age categories were targeted: children (5–13 years), teenagers (14–17 years) and adults (>18 years). For the two pathological populations (CP and OMD), a representative sample of the patients generally coming to the laboratory for CGA was included. The general and group-specific inclusion and exclusion criteria are detailed in the Supplementary Materials (Table S1).

Sample size justification:

A minimum of 12 participants in the AS group, 10 participants in the CP group and 24 in the OMD group were determined based on the concurrent validity objective for this study. The sample sizes were calculated using the means and standard deviations of the RMSE found in the literature assessing an IMU system against an optoelectronic system. The power was set to 0.95, and α to 0.05. The calculation was based on the following nine outcomes that were reported by previous studies: the hip, knee and ankle angles in flexion/extension, abduction/adduction and internal/external rotations. Two reference studies [13,14] were used to compute the sample size for the AS group (maximal sample size computed = 12 among all outcomes and studies), and a reference study [15] was used to compute the sample size of the CP group (maximal sample size computed = 10 among all outcomes). Since no reference values were found for other pathological populations, we decided to double the sample size of the AS group for the OMD group.

### 2.3. Equipment

**Figure 1:**
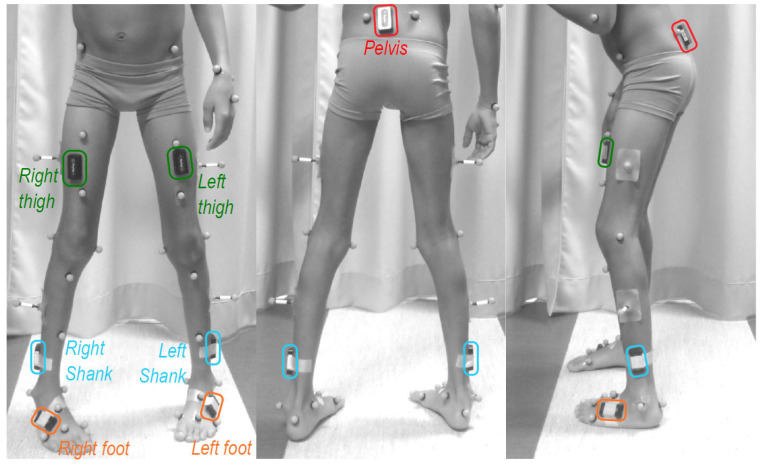
Equipment on the participants including 7 IMUs on the pelvis (red), thighs (green), shanks (blue) and feet (orange), and 34 reflective markers placed following the Conventional Gait Model 1.1.

The equipment was installed by one operator. Participants were simultaneously measured by a 7-IMU (Physilog5, GaitUp, Renens, Switzerland) inertial system at 256 Hz and a twelve-camera (Oqus7+) optoelectronic system (Qualisys, Göteborg, Sweden) at 100 Hz. The IMUs were positioned on the lower back (at sacrum level, in the middle and below the posterior superior iliac spines), the thighs (on the frontal side), the shanks (on the distal lateral side) and the feet (on the top) (Figure 1). Tridimensional acceleration and angular velocity were acquired with ranges of ±16 g and ±2000°/s respectively. Reflective markers were placed according to the Conventional Gait Model 2.4 (CGM 2.4) [16] on the lower limbs (Figure 1).

### 2.4. Protocol

At the beginning and the end of each measurement session, a synchronization (‘sync’) trial was performed. During this trial, the acceleration signal from one Physilog IMU and the trajectory of a reflective marker, both attached to a rigid wand, were recorded simultaneously while the wand was dropped onto the ground. The resulting impact generated a distinct acceleration peak in the IMU signal and a clear event in the optoelectronic trajectory data. These ‘sync’ trials were used to temporally align the inertial and optoelectronic systems as no hardware synchronization was available.

Each participant was asked to stand still in a neutral pose with the legs as vertical as possible and parallel feet, then to walk back and forth along the 10 m walkway at spontaneous speed like during a classical CGA session.

For the first session, the protocol was led by one operator (A). For the second session, the protocol was led first by one operator (A) and then, after equipment removal and 15 min rest, the protocol was repeated and led by another operator (B).

### 2.5. Data Processing

**Figure 2:**
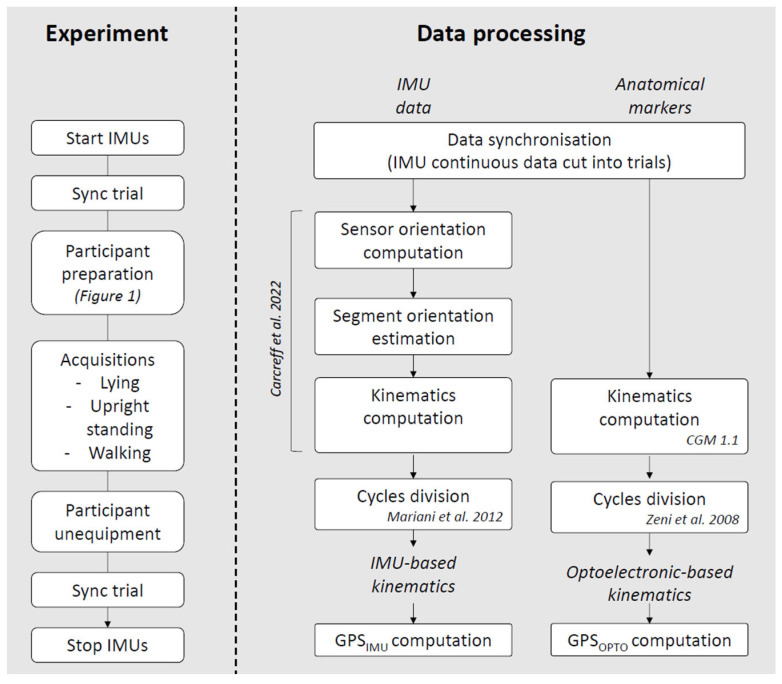
Flowchart of the experimental procedure and data processing main steps. IMUs: inertial measurement units, CGM: Conventional Gait Model; GPS: Gait Profile Score; OPTO: optoelectronic. References: Carcreff et al., 2022 [19], CGM1.1 [16], Mariani et al., 2012 [20], Zeni et al., 2008 [18].

Data processing and analysis were performed using Matlab R2018b software (Mathworks, Natick, MA, USA) and R v.4.1.1 and the RStudio interface (v.1.4.1717, Rstudio Team) following the workflow presented in Figure 2.

Synchronization between the optoelectronic and IMU systems was performed offline during post-processing using the ‘sync’ trials described above. The impact event generated during the wand drop was identified in both the IMU acceleration signal and the optoelectronic marker trajectory. The absolute timestamps of these events were then used to compute the temporal offset between systems. Following synchronization, inertial data were segmented into individual gait trials according to the optoelectronic trial timestamps, ensuring temporal consistency between both data streams for all subsequent kinematic analyses. The gaps in the marker trajectories were automatically filled using information of inter-correlated markers obtained from a principal component analysis [17]. Gait events (foot strikes and foot offs) were computed from the feet and pelvis marker trajectories as proposed by Zeni et al. [18] and checked manually.

Sensor orientation estimation, sensor-to-segment alignment and kinematics computation were performed as described in Carcreff et al. [19], with the exception of the sitting trial which, in the current study, was replaced by a lying trial for the same purpose (mediolateral axis correction). This lying trial was performed at the beginning of each measurement session, asking the participant to lie still on a medical table with legs extended and feet facing the ceiling.

The resulting kinematics data was cut into gait cycles, using the events detected on the raw inertial data as proposed by Mariani et al. [20] for the IMU data. The reference kinematic data was computed using the CGM 1.1 [16]. The marker set used during acquisition was compatible with the PyCGM2.x framework including CGM 2.4 (Figure 1); however, joint kinematics were computed using the CGM 1.1 model, chosen for its widespread clinical use and status as the reference standard. The additional markers were included to enhance trajectory reconstruction and ensure complete data for CGM 1.1 calculations.

### 2.6. Outcomes

**Figure 3:**
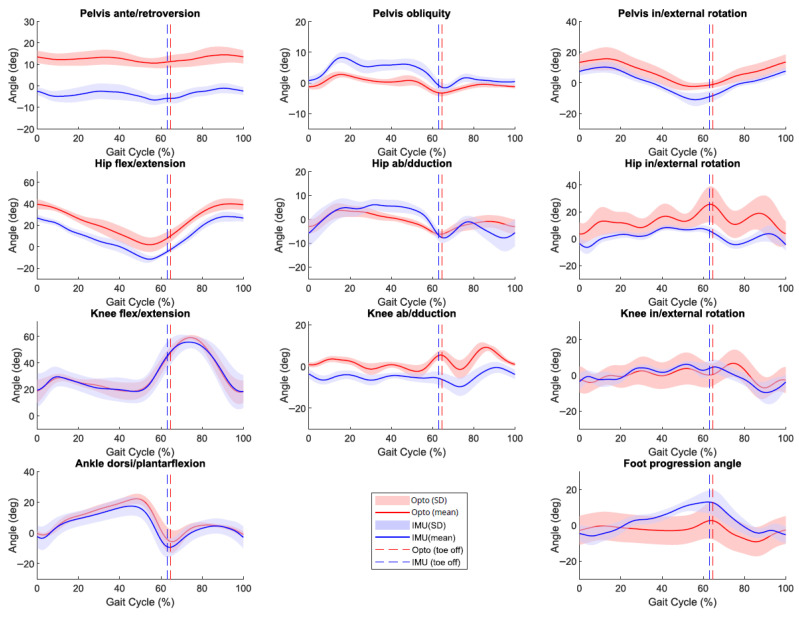
Mean (and standard deviation (SD)) kinematics of the right side of one study participant with cerebral palsy computed from the optoelectronic (red) and IMU (blue) systems across several gait trials.

The 11 most commonly reported gait kinematic outcomes [21] were computed: pelvis ante/retroversion, pelvis obliquity, pelvis in/external rotations, hip flex/extension, hip ab/adduction, hip in/external rotations, knee flex/extension, knee ab/adduction, knee in/external rotations, ankle dorsi/plantar flexion, and foot progression angle (Figure 3).

The Gait Profile Scores (GPS) were also computed in order to have an index of the gait pattern quality [22]. It consists of the direct root mean square distance between an individual’s data and the mean normal data calculated across the gait cycle for nine joint angles (pelvis and hip angles in three planes, knee and ankle angles in the sagittal plane and foot progression) of one side [22]. The higher the GPS (°), the more affected the gait pattern gets, with a normal value approaching 5° [22]. The GPS computed from optoelectronic data (GPS_OPTO_) and the GPS computed from the IMU data (GPS_IMU_) were reported separately. For the GPS_OPTO_, the mean normal data was the mean kinematics of the AS group computed with the optoelectronic system. For the GPS_IMU_, the mean normal data was the mean kinematics of the AS group computed with the IMU system.

### 2.7. Data Analysis

#### 2.7.1. A-Concurrent Validity

The concurrent validity was assessed for the first session only, through the RMSE, the RMSE centered at the mean (as proposed in Nuesch et al. [23]), the Pearson’s correlation coefficient (CC) and the absolute difference in range of motion (ΔROM) between the optoelectronic-based and the IMU-based kinematics. These validity metrics were computed cycle by cycle and then averaged across sides and gait trials. The results were separated by groups. According to CGA standards, errors below 5° were considered as clinically acceptable, in line with the intrinsic accuracy limits of optoelectronic motion capture systems [24].

Furthermore, the link between the IMU system’s errors and the level of disability was assessed through Pearson’s CCs between the RMSEs and the mean of right and left GPS_OPTO_ for all the study participants. Altman’s guidelines were used to interpret the correlations: poor, if CC < 0.20; fair, if 0.20 ≤ CC < 0.40; moderate, if 0.40 ≤ CC < 0.60; good, if 0.60 ≤ CC < 0.80; and very good, if CC ≥ 0.80 [25].

CCs were also computed between the RMSE and the GPS_OPTO_ to investigate whether the errors were linked to the participant’s level of gait disorder.

#### 2.7.2. B-Reliability

The reliability was assessed through intraclass correlation coefficient (ICC). For its calculation, the variance of ‘class’ components [26], associated with the session and the operator, were computed from a linear two-way mixed effects model [27] with the lme4 package of R [28]. The intra-operator reliability was assessed between the first and the second sessions, one week apart, led by one operator (A). ICCs were computed for discrete parameters including the maximal, minimal and mean values of the kinematic curves which correspond to clinically relevant events [29]. The inter-operator reliability was assessed between the two operators (A and B) during the second session. In line with COSMIN recommendations, Koo and Li’s guidelines were used to read ICC scores: poor reliability for ICC < 0.50, moderate reliability for 0.50 ≤ ICC < 0.75, good reliability for 0.75 ≤ ICC < 0.90 and excellent reliability for ICC ≥ 0.90 [30].

#### 2.7.3. C-Interpretability

The interpretability was assessed through the comparison between the GPS_OPTO_ and the GPS_IMU_ (averaged by sides) using a paired student t-test and Pearson correlation for normally distributed data, and a paired Wilcoxon test and Spearman correlation for non-normally distributed data. Comparisons between systems (OPTO-IMU) for each group were conducted using paired Wilcoxon tests with Bonferroni correction. Group comparisons were performed using an appropriate analysis of variance with pairwise comparisons.

Interpretation was based on previously reported minimal detectable changes (MDCs): 1.7° for the CP and OMD groups [31,32], and 0.7° for the AS group [33].

## 3. Results

Fifty-five participants were included in total in this study: 15 in the AS group, 15 in the CP group and 25 in the OMD group. Among the OMD group, the main clinical profiles were club foot, idiopathic toe walking, drop foot, muscular dystrophia, torsional troubles, poliomyelitis, ataxia, and foot deformities. The distribution across age subgroups is presented in Table 1.

A table with all the computed kinematic outcomes is available in an open access repository (Zenodo) [34].

### 3.1. A-Concurrent Validity

**Figure 4:**
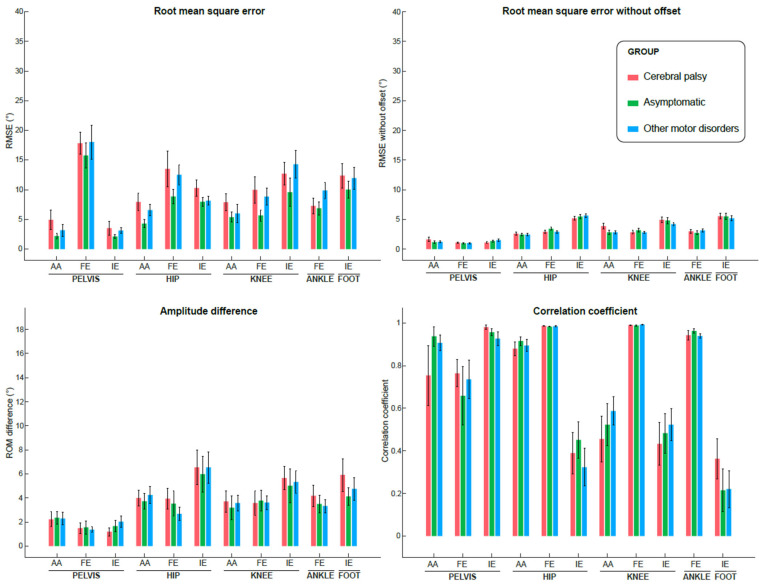
Metrics (root mean square error (RMSE), RMSE centered at the mean, correlation coefficient, and difference in range of motion (ROM)) of validity evaluation of the IMU method against the optoelectronic reference for each group. Histograms represent the mean values (and interval confidence bars) for all participants, for each joint/segment, and for each plane. AA: obliquity–ab/adduction; FE: ante/retroversion–flex/extension; IE: in/external rotation–progression angle.

The validity metrics per kinematic outputs and groups are depicted in Figure 4 and the corresponding numeric values are available in the Supplementary Materials (Table S2).

The mean RMSEs across all kinematic outcomes were 7.1° for the AS group, 9.8° for the CP group and 9.3° for the OMD group, with maximal errors found at the pelvis ante/retroversion level and minimal errors found at the pelvis rotation level for the three groups. These errors corresponded on average to about 0.8–0.9% (with a maximum of 5.5%) of the kinematic curve amplitude (‘Relative RMSE’ in Table S2).

The mean centered RMSEs, with offset removal, were 3.1° for the AS group, 3.1° for the CP group and 3.0° for the OMD group. The highest errors were then found at the foot progression angle and hip rotation levels.

Overall good correlations were found across all outcomes and groups (mean CC between 0.72 and 0.73). Very good correlations were found for the three groups in the sagittal plane (hip, knee and ankle), as well as in the pelvis rotation and hip ab/adduction (CC > 0.8). The poorest correlations were found for the hip rotation (CC: [0.32–0.45]), knee ab/adduction (CC: [0.46–0.59]) and rotation (CC: [0.43–0.52]), and foot progression angle (CC: [0.22–0.36]).

The means ΔROM across all outcomes were 3.5° for the AS group, 3.8° for the CP group and 3.6° for the OMD group.

Correlations between the RMSE and the GPS_OPTO_ (Table 2) ranged from poor (CC = 0.15 for knee ab/adduction) to moderate (CC = 0.51 for pelvis rotation), indicating a poor link between measurement error and the level of disability.

### 3.2. B-Reliability

Thirty-one individuals participated in the second measurement session: fifteen from the AS group (same as the first session), eight from the CP group (five children, one teenager, and two adults), and eight from the OMD group (four children, two teenagers, and two adults). Due to technical issues with the sensors (e.g., synchronization between inertial sensors, incorrect handling, battery loss), only 27 participants (13 AS, 7 CP, 7 OMD) were included in the reliability analysis.

Table 3 reports the intra- and inter-operator ICCs of the IMU system computed for four discrete parameters (maximal, minimal values, range of motion (ROM), and the mean over gait cycles), as well as those of the reference system. A more complete table (with more parameters tested and with more statistical outcomes reported) is available in the Supplementary Materials (Table S3). From poor to good intra-operator reliability was found for the IMU-based kinematics in the sagittal and frontal planes (minimal ICC = 0.24 for the knee ab/adduction ROM; maximal ICC = 0.82 for the mean pelvis obliquity). Mainly poor intra-operator reliability was found in the transverse plane (ICCs ranging from 0.13 for the mean pelvis rotation to 0.53 for the foot progression angle ROM). From moderate to good inter-operator reliability was found in the sagittal and frontal planes (from 0.53 for the minimum hip flexion/extension to 0.85 for the maximum knee flexion/extension) and poor to moderate in the transverse plane (from 0.08 for the minimum pelvis rotation to 0.68 for the hip rotation ROM) (Table 3).

Intra-operator ICCs for kinematic variables obtained from the reference system across joints and planes ranged from 0.30 to 0.84, and inter-operator ICCs ranged from 0.46 to 0.85.

### 3.3. C-Interpretability

The normality of GPS_OPTO_ and GPS_IMU_ distributions was visually assessed using histograms. Non-parametric tests were applied due to the uneven distribution of GPS_OPTO_ across the two pathological groups. No statistical difference was found between both scores for the pathological groups (*p* = 0.84), and a significant moderate correlation (CC = 0.42, *p*-value = 0.007) was found. Figure 3 and Table 4 detail the scores for the groups.

## 4. Discussion

The aim of this study was to evaluate the concurrent validity (A), reliability (B) and interpretability (C) of a previously described IMU-based 3D lower limb kinematics computational method [19] on asymptomatic individuals and patients with heterogeneous gait disorders. Concurrent validity was generally found acceptable (RMSE < 10°, good correlations and ΔROM < 5°) for all groups of subjects in the three planes. However, important exceptions must warrant attention such as the pelvis ante/retroversion which resulted in a noteworthy offset (constant error throughout the gait cycle) of about 15°. The errors were not associated with the severity of the gait deviations. Intra- and inter-operator reliability were highly dependent on the chosen discrete parameter and the plane assessed (from poor to good reliability). Finally, the proposed IMU-based system could be used to compute gait quality scores such as the GPS since it was not found different from the one computed from the optoelectronic reference.

### 4.1. A-Concurrent Validity

The results obtained in the current study were similar to and even better than the results obtained in our previous paper discussing the concurrent validity of the IMU system against the optoelectronic reference for asymptomatic individuals only [19]. Indeed, for the AS group, we found a mean RMSE of 7.1° across all joints and planes, while 7.5° was previously found, a mean centered RMSE of 3° while 4° previously, a mean correlation coefficient of 0.73, while 0.63 previously, and a mean ΔROM of 3.5° while 6.8° previously. A possible explanation for these slight differences between both studies is the different locations of the IMUs on the body segments (e.g., frontal side of the thighs instead of lateral, lateral and distal side of the shank instead of medial and proximal). Just like reflective markers, IMUs suffer from soft tissue artifacts. A recent study has demonstrated for instance that an IMU placed on the frontal side of the thigh gives better stability than an IMU located on the lateral side of the thigh [7]. The better results found with the current sensor placement might confirm this statement.

Except for the pelvis ante/retroversion, the present results seemed comparable to existing studies. In the meta-analysis of Kobsar et al. [35], RMSEs ranged between 0.7° and 11.2° across 14 studies assessing the validity of 3D kinematic outcomes in healthy individuals. In pathological populations, there are few existing studies, and making comparisons is challenging due to disparities in sensor placement, reference systems, methodologies employed for kinematic calculations, and the diversity of reported outcomes. Prisco et al. recently reviewed the literature for the concurrent validity of wearables for gait analysis [36]. In this review, none of the studies which included pathological populations (10/32) studied kinematics.

The difference between the asymptomatic and the pathological (CP and OMD) individuals regarding the concurrent validity of the IMU system was only revealed by the RMSE values. A higher mean RMSE of about 2.5° was indeed found in the CP and OMD groups, but centered RMSE, correlation coefficients and ROM differences were very close to the AS group, meaning that the kinematic waveforms were well respected in the three groups. The main difference was thus provided by the kinematic offset, i.e., constant angle separating the IMU-based kinematic curve from the optoelectronic-based kinematic curve. This offset is a well-known issue while computing gait kinematics from IMUs [13,23,37,38,39] and is often related to the difference in biomechanical models, with different definitions of the anatomical framework [38,39]. While the optoelectronic system determines the segment frames thanks to the anatomical points located by cutaneous markers, the IMU system constructs the segment frames from functional axes determined by calibration postures or movements [38]. To address this issue, Ortigas Vàsquez et al. [40] used a robotic simulator driven by real-world data to evaluate the accuracy of their IMU system in computing 3D knee kinematics. The resulting errors of their tested IMU system were significantly smaller (RMSE ≤ 0.9°, maximum absolute error ≤ 3.2°). The kinematic offset can also be attributed to the lack of knowledge of the initial position of the IMU sensors. In general, participants perform predefined calibration postures and movements to enrich the knowledge of sensor positions related to the reference frame and/or the underlying body segments. In our method, these calibration postures and movements were kept to a minimum to be feasible for patients with motor disorders. This is the main reason why a substantial constant offset was observed at the pelvis level. No specific calibration posture or movement was required to align the pelvic-mounted sensor with the anatomical pelvis in the sagittal plane. As a result, the natural sacral inclination was implicitly considered as the neutral pelvic orientation, which differs from the definition used in the Conventional Gait Model. This methodological choice primarily affects absolute pelvic angle values, while preserving the overall waveform shape and temporal characteristics.

From a clinical perspective, this limitation may restrict the use of absolute pelvic ante/retroversion values for diagnostic interpretation or inter-subject comparison. However, the preserved waveform consistency suggests that IMU-based pelvic kinematics remain relevant for within-subject analyses, longitudinal follow-up, and the assessment of changes induced by therapeutic interventions.

### 4.2. B-Reliability

The intra- and inter-operator reliability provided by our IMU system were poor to good depending on the chosen discrete kinematic parameter and the plane. Overall, the reliability of the system was found better for the sagittal and frontal kinematics, which is in accordance with previous studies [11,35]. However, our ICC values appeared lower than previously published values. This may be explained by the inclusion of pathological subjects instead of only asymptomatic individuals [29,38,41]. Inter-operator reliability was found higher than intra-operator reliability. This could be explained by the intrinsic gait variability between two visits (one week apart) [42]. Finally, higher ICCs were mostly found for the reference optoelectronic system, especially at the hip and knee levels in the sagittal plane, and the pelvis and the foot level in the transverse plane. Intra-operator ICCs for kinematic variables obtained from the IMU system ranged from 0.13 to 0.82 across joints and planes, and ICCs obtained from the reference system ranged from 0.30 to 0.84. Inter-operator ICCs obtained from the IMU system ranged from 0.06 to 0.85, and those obtained from the reference ranged from 0.46 to 0.85. Lower reliability values were predominantly observed for transverse-plane kinematics, particularly at the pelvis and foot levels. From a clinical standpoint, this finding indicates that the use of an IMU system for transverse-plane kinematics may be more appropriate for descriptive or exploratory analyses rather than precise clinical decision-making.

### 4.3. C-Interpretability

To be incorporated into clinical practice, gait analysis outcomes need to guarantee a high level of interpretability (the degree to which one can assign qualitative meaning [12]). We found that one of the most commonly used gait scores, the GPS, used to summarize the gait deviation level, was correctly estimated from the IMU system as compared to the optoelectronic system for both pathological groups. Surprisingly, this was not the case for the AS group. This discrepancy may be explained by the difference in heterogeneity between the pathological and the asymptomatic groups. Pathological participants show larger and more heterogeneous gait deviations, making the GPS less sensitive to small measurement differences, whereas asymptomatic participants have low and homogeneous GPS values (centered around 5°), where minor methodological biases can lead to statistically significant differences. These findings suggest that while IMU-based GPS estimates are appropriate for summarizing gait deviations in populations with motor disorders, their interpretability may be reduced in asymptomatic individuals.

**Figure 5:**
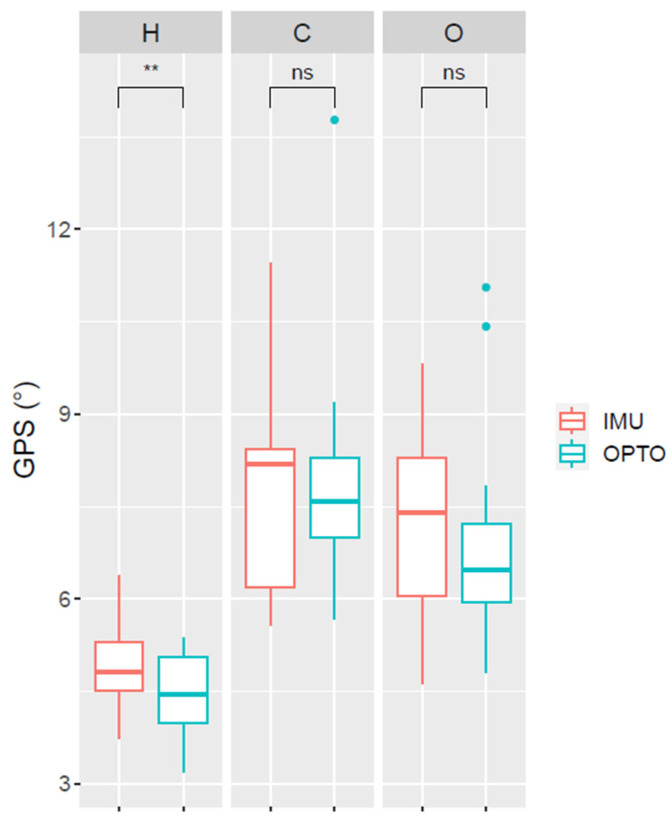
Mean left and right Gait Profile Scores (GPS) (°) computed from inertial (IMU) and optoelectronic (OPTO) data for the 3 groups (H: asymptomatic, C: cerebral palsy, O: other motor disorders); ns: non-significant difference; **: significant difference. The three outliers highlighted in Figure 5 based on optoelectronic data correspond to participants with severe gait impairments (bilateral CP with crouch gait, unilateral drop foot, and excessive external rotation of feet and hips). They were not identified as outliers in the IMU-based kinematic outcomes.

Additionally, individuals identified as outliers based on optoelectronic GPS values (GPS_OPTO_; Figure 5) were not identified as outliers when using the IMU-based method, suggesting an attenuation of large kinematic deviations relative to the reference system. This finding highlights a potential limitation of our IMU-based kinematic analysis in capturing extreme gait deviations. To the best of our knowledge, no study evaluated a similar interpretability metric. Further analysis is needed to explore the interpretability in greater depth, including a larger sample size.

### 4.4. Limitations

The patients included in this study were not selected based on their walking capacities or motor disorders. They were included as they came for a CGA, provided they were willing to participate and met the inclusion criteria. This may explain the relatively low impairment degree in the two pathological subgroups, and may partly explain the equivalence in the method’s performance for pathological groups as compared to the healthy group. Another limitation of this study was the use of different gait events (foot strikes and foot offs) for the two compared methods (IMU and OPTO). Errors in gait event detection could thus have led to errors in the kinematics; however, this choice was made to be the closest to the future conditions and processes (IMUs alone).

### 4.5. Perspectives

Our study highlighted the potential of wearable technology in gait analysis, offering promising applications for low-resource laboratories and clinics. However, several challenges have been identified and discussed in the preceding sections. Computing gait kinematics with IMUs remains complex, and integrating AI into algorithms should enhance the accuracy and reliability of the results, as demonstrated by several studies [43,44].

Three-dimensional kinematic computations, similar to those performed by optoelectronic systems in laboratories, may not be the optimal application for IMU-based systems. Instead, their greatest value lies in their usability outside laboratory settings, particularly for long-term and real-world monitoring [45]. Future efforts should focus on reducing the number of sensors required without compromising accuracy, making these systems less obtrusive and more practical.

Finally, given the challenges of 3D kinematics computation with IMUs, markerless motion capture methods, bolstered by advancements in AI, could surpass wearable sensors in specific applications [46]. In fact, rather than viewing these technologies as competitors, they should be seen as complementary. Optoelectronic systems excel in providing highly detailed and accurate biomechanical data in controlled environments. IMU-based systems are ideal for real-world, long-duration measurements, while markerless methods offer significant potential for efficient screening and assessment. The choice of technology should therefore be guided by the specific context and objectives of the analysis [46].

## 5. Conclusions

This study demonstrates the validity, reliability, and interpretability of IMU-based systems for estimating 3D lower limb kinematics in both asymptomatic individuals and populations with gait disorders. The findings highlight the potential of IMU-based methods as a viable alternative to optoelectronic systems in clinical and resource-limited settings. Despite the observed limitations in specific parameters, such as pelvis ante/retroversion, the good agreement between the kinematic waveforms, strong agreement in Gait Profile Scores and acceptable reliability across most metrics underscore the relevance of this approach. The acquired kinematic data can be used to objectively characterize gait impairments, support clinical decision-making, and monitor changes in gait patterns over time, particularly in contexts where conventional gait laboratories are not accessible. Such data may also facilitate large-scale or longitudinal studies by enabling repeated gait assessments in ecological environments. Future research should focus on optimizing sensor alignment and expanding applications to enhance the precision and versatility of IMU-based gait analysis.

## Figures and Tables

**Table 1:** Participants’ characteristics.

Group		Asymptomatic (n = 15)			Cerebral Palsy (n = 15)			Other Motor Disorders (n = 25)		
Age Group		Children (n = 4)	Teenagers (n = 5)	Adults (n = 6)	Children (n = 8)	Teenagers (n = 5)	Adults (n = 2)	Children (n = 13)	Teenagers (n = 3)	Adults (n = 9)
Age (years)	mean	9.0	16.0	32.4	10.1	15.0	18.5	9.2	15.3	42.2
	SD	1.4	1.0	5.7	1.5	0.9	0.5	2.2	0.5	20.2
Height (cm)	mean	135.1	169.9	165.3	141.8	166.8	167.8	140.3	163.7	166.3
	SD	10.4	8.6	10.6	7.2	8.4	0.8	14.8	7.6	11.3
Weight (kg)	mean	30.4	58.7	65.6	36.1	56.1	68.3	39.0	55.5	71.1
	SD	7.1	13.8	17.4	5.7	16.2	10.3	14.7	4.8	12.7
BMI (kg/m^2^)	mean	16.4	20.1	23.5	17.9	19.8	24.2	19.0	20.8	25.6
	SD	2.0	3.4	3.2	2.2	3.8	3.4	4.0	1.5	2.7
GMFCS	n				I—5 II—2 III—1	I—4 III—1	I—2			
GPS (°)	mean	5.0	4.0	4.4	8.1	8.0	7.4	6.6	8.6	6.6
	SD	0.4	0.8	0.5	2.4	1.7	1.8	0.6	2.6	1.2

BMI: Body Mass Index; GMFCS: Gross Motor Classification System; GPS: Gait Profile Score.

**Table 2:** Correlations between the root mean square error (RMSE) and the Gait Profile Score (GPS).

Kinematic Outcomes	Pearson’s Correlation Coefficient	*p*-Value
Pelvis ante/retroversion	0.26	0.053
Pelvis obliquity	0.17	0.204
Pelvis rotation	0.51	<0.001
Hip flexion/extension	0.38	<0.001
Hip ab/adduction	0.32	0.001
Hip rotation	0.21	0.025
Knee flexion/extension	0.46	<0.001
Knee ab/adduction	0.15	0.108
Knee rotation	0.23	0.014
Ankle flexion/extension	0.19	0.047
Foot progression angle	0.23	0.014

**Table 3:** Intra- and inter-operator intraclass correlation coefficients (ICCs) for both inertial (IMU) and reference optoelectronic (OPTO) systems for 4 discrete parameters (maximal, minimal values, range of motion (ROM) and the mean over gait cycles). Color gradient from excellent (green) to poor (red) reliability according to Koo & Li [30].

Plane	Segment/Joint	Parameter	Intra-Operator ICC		Inter-Operator ICC	
			IMU	OPTO	IMU	OPTO
Sagittal	Pelvis	Max	0.40	0.82	0.57	0.85
		Min	0.49	0.67	0.56	0.77
		ROM	0.76	0.78	0.84	0.80
		Mean	0.39	0.77	0.50	0.82
	Hip	Max	0.63	0.71	0.59	0.81
		Min	0.37	0.78	0.53	0.85
		ROM	0.71	0.77	0.68	0.79
		Mean	0.56	0.69	0.57	0.82
	Knee	Max	0.82	0.62	0.85	0.62
		Min	0.67	0.49	0.72	0.39
		ROM	0.60	0.75	0.74	0.76
		Mean	0.69	0.49	0.77	0.44
	Ankle	Max	0.51	0.73	0.61	0.75
		Min	0.50	0.65	0.66	0.76
		ROM	0.68	0.73	0.72	0.76
		Mean	0.45	0.71	0.57	0.75
Frontal	Pelvis	Max	0.81	0.68	0.77	0.65
		Min	0.72	0.63	0.70	0.65
		ROM	0.69	0.79	0.70	0.82
		Mean	0.82	0.49	0.80	0.51
	Hip	Max	0.63	0.70	0.69	0.74
		Min	0.69	0.55	0.70	0.72
		ROM	0.63	0.67	0.66	0.76
		Mean	0.69	0.63	0.75	0.69
	Knee	Max	0.58	0.28	0.73	0.60
		Min	0.70	0.48	0.71	0.58
		ROM	0.24	0.32	0.55	0.46
		Mean	0.68	0.50	0.71	0.78
Transverse	Pelvis	Max	0.35	0.68	0.39	0.68
		Min	0.40	0.46	0.08	0.49
		ROM	0.50	0.56	0.17	0.50
		Mean	0.13	0.59	0.06	0.60
	Hip	Max	0.22	0.35	0.44	0.64
		Min	0.49	0.30	0.70	0.59
		ROM	0.47	0.37	0.68	0.63
		Mean	0.36	0.31	0.54	0.55
	Knee	Max	0.51	0.49	0.60	0.58
		Min	0.39	0.50	0.42	0.66
		ROM	0.50	0.39	0.56	0.65
		Mean	0.42	0.48	0.58	0.58
	Foot Progression Angle	Max	0.52	0.81	0.60	0.84
		Min	0.29	0.73	0.28	0.83
		ROM	0.53	0.67	0.57	0.69
		Mean	0.29	0.82	0.37	0.88

**Table 4:** Mean (and standard deviation) of the mean left and right Gait Profile Scores (GPS) (°) computed from inertial (IMU) and optoelectronic (OPTO) data for the 3 groups.

	GPS_OPTO_	GPS_IMU_	Systems Comparisons ^c^
Asymptomatic (n = 15)	4.4 (0.7)	4.9 (0.7)	*p* = 0.015
Cerebral palsy (n = 15)	8.0 (1.9)	7.7 (1.7)	*p* = 0.720
Other motor disorders (n = 25)	6.8 (1.4)	7.0 (1.5)	*p* = 0.491
Groups comparisons ^a^	*p* < 0.001	*p* < 0.001	
Pairwise comparisons with asymptomatic group ^b^	CP vs. TD: *p* < 0.001 OMD vs. TD: *p* < 0.001	CP vs. TD: *p* < 0.001 OMD vs. TD: *p* < 0.001	

^a^: Kruskall–Wallis ANOVA; ^b^: Wilcoxon unpaired tests with Bonferroni adjustment; ^c^: Wilcoxon paired tests.

## Data Availability

The data presented in the study are openly available in Zenodo at https://doi.org/10.5281/zenodo.18068953.

## Supplementary Materials

The following supporting information can be downloaded at https://www.mdpi.com/article/10.3390/s26061746/s1, Table S1: Inclusion and exclusion criteria; Table S2: IMU-based kinematics validity against the optoelectronic-based kinematics.; Table S3: Reliability results including intraclass correlation coefficients (ICCs), standard errors of measurement (SEMs) and minimal detectable changes (MDCs).

## Abbreviations

The following abbreviations are used in this manuscript:

AS	Asymptomatic
CC	Correlation coefficient
CGA	Clinical gait analysis
CGM	Conventional Gait Model
CP	Cerebral palsy
GMFCS	Gross motor function classification system
GPS	Gait Profile Score
HR-PRO	Health related-patient reported outcomes
ICC	Intraclass correlation coefficient
IMU	Inertial measurement unit
OMD	Other motor disorders
OPTO	Optoelectronic
RMSE	Root mean square error
ROM	Range of motion
