# Supplementary material for: Validity, Reliability and Interpretability of an IMU-Based System to Measure 3D Lower Limb Kinematics of Patients with Heterogeneous Gait Disorders
Source: Sensors (Basel). 2026 Mar 10;26(6):1746. doi: 10.3390/s26061746 (PMC13030621; doi:10.3390/s26061746)
Supplement: Supplementary file 1 [file sensors-26-01746-s001.zip › Supplementary material captions.pdf]

Supplementary material captions

Supp_table S1 - Inclusion and exclusion criteria

Supp_table S2– IMU-based kinematics validity against the optoelectronic-based kinematics.
Legend: RMSE: root mean square error; ROM: range of motion; AS: asymptomatic; CP: cerebral palsy; OMD: Other motor disorders; sd: standard deviation

Supp_table S3 - Reliability results including intraclass correlation coefficients (ICC); standard errors of measurement (SEM) and minimal detectable changes (MDC).
Assessed variables: Stride time, stride length, walking speed, percentage of stance phase, maximal value of the kinematic curve over the gait cycle, minimal value, range of motion, mean value, standard deviation of the kinematic curve, maximal value in stance phase, minimal value in stance phase, range of motion in stance phase, mean value in stance phase, standard deviation in stance phase, maximal value in swing phase, minimal value in swing phase, range of motion in swing phase, mean value in swing phase, standard deviation in swing phase, and values at each frame of the gait cycle (101 frames).

Legend: fe: flexion/extension; aa: ab/adduction; ie: internal/external rotation; I: inertial measurement units (IMU); O: optoelectronic system; ROM: Range of motion; Std: standard deviation; intra_Sess: intra-session; inter_Sess: inter-session; intra_Ope: intra-operator; inter_Ope: inter-operator.
