# Supplementary material for: Validity, Reliability and Interpretability of an IMU-Based System to Measure 3D Lower Limb Kinematics of Patients with Heterogeneous Gait Disorders
Source: Sensors (Basel). 2026 Mar 10;26(6):1746. doi: 10.3390/s26061746 (PMC13030621; doi:10.3390/s26061746)
Supplement: Supplementary file 1 [file sensors-26-01746-s001.zip › TableS1.pdf]

Supp_table 1 - Inclusion and exclusion criteria

	INCLUSION	EXCLUSION
**GENERAL**	Willing to participate and able to provide informed consent signed by a legal tutor	• Previous medical history of lower-limb arthroplasty/fusion or spine surgery<br>• Previous surgery on the last 12 months<br>• Pregnancy if known<br>• Known allergy to hypoallergenic adhesive tape
**AS GROUP**		Muscular skeletal, or other diseases significantly influencing gait
**CP GROUP**	• Diagnostic of cerebral palsy classified between GMFCS I, II, or III<br>• Ability to walk 10 meters without walking aid	
**OMD GROUP**	Diagnostic of motor disease significantly influencing gait	

AS: Asymptomatic; CP: Cerebral palsy; OMD: Other motor disorders
